# Dietary glycaemic index and glycaemic load in relation to risk of breast cancer

**DOI:** 10.1017/S1368980021004018

**Published:** 2022-06

**Authors:** Somaye Rigi, Asma Salari-Moghaddam, Sanaz Benisi-Kohansal, Leila Azadbakht, Ahmad Esmaillzadeh

**Affiliations:** 1 Department of Community Nutrition, School of Nutritional Sciences and Dietetics, Tehran University of Medical Sciences, Tehran 14155-6117, Iran; 2 Diabetes Research Center, Endocrinology and Metabolism Clinical Sciences Institute, Tehran University of Medical Sciences, Tehran, Iran; 3 Obesity and Eating Habits Research Center, Endocrinology and Metabolism Molecular-Cellular, Tehran University of Medical Sciences, Tehran, Iran; 4 Department of Community Nutrition, School of Nutrition and Food Science, Isfahan University of Medical Sciences, Isfahan, Iran

**Keywords:** Glycaemic index, Glycaemic load, Breast cancer, Case–control study

## Abstract

**Objective:**

Previous studies on the association between glycaemic index (GI) and glycaemic load (GL) in relation to breast cancer risk are contradictory. The aim of this study was to examine the association between dietary GI and GL and risk of breast cancer in Iranian women.

**Design:**

Population-based case–control study. Dietary GI and GL were assessed using a validated Willett-format 106-item semi-quantitative FFQ.

**Setting:**

Isfahan, Iran.

**Participants:**

Cases were 350 patients with newly diagnosed stage I–IV breast cancer, for whom the status of breast cancer was confirmed by physical examination and mammography. Controls were 700 age-matched apparently healthy individuals who were randomly selected from general population.

**Results:**

After controlling for potential confounders, individuals in the highest tertile of dietary GI had 47 % higher odds of breast cancer than women in the lowest tertile (OR: 1·47; (95 % CI 1·02, 2·12)). Stratified analysis by menopausal status showed such association among postmenopausal women (OR: 1·51; (95 % CI 1·02, 2·23)). We found no significant association between dietary GL and odds of breast cancer either before (OR: 1·35; (95 % CI 0·99, 1·84)) or after adjustment for potential confounders (OR: 1·24; (95 % CI 0·86, 1·79)). In addition, stratified analysis by menopausal status revealed no significant association between dietary GL and odds of breast cancer.

**Conclusions:**

Our findings showed a significant positive association between dietary GI and odds of breast cancer. However, we observed no significant association between dietary GL and odds of breast cancer.

Breast cancer (BC) is the second most common cancer globally that affects 1·4 million subjects each year^(1,2)^. In Iran, among different types of cancer, breast cancer is considered as the first malignancy diagnosed in women^(3)^.

The aetiology of breast cancer is largely unknown. However, several factors including age, sex, race, genetic factors, reproductive history, menopausal hormone use, alcohol intake, tobacco use, physical inactivity, radiation exposure and dietary intakes might contribute to its aetiology^(4)^. Although the majority of studies assessing the association between diet and breast cancer have emphasised on the role of dietary fats, dietary carbohydrate intake may also play a role in this regard. Dietary glycaemic index (GI) and glycaemic load (GL), as indicators of quality and quantity of dietary carbohydrate intake^(5)^, have extensively been investigated in relation to risk of breast cancer in earlier studies; however, findings are contradictory. For instance, dietary GL was positively associated with the risk of breast cancer in the Italian European Prospective Investigation into Cancer and Nutrition cohort; however, such association was not observed for dietary GI^(6)^. In a cohort study among Canadian postmenopausal women, high-GI diet was associated with an increased risk of breast cancer^(7)^. However, no significant association was observed between dietary GI and GL and the risk of breast cancer in another prospective study^(8)^.

Most previous studies on the association between dietary GI, GL and risk of breast cancer came from developed countries and limited information is available in developing countries in this regard. Investigating the association of dietary GI and GL and the risk of breast cancer is particularly relevant for the Middle-Eastern countries, where more than 60 % of total energy intake is taken from carbohydrates and much of them are high GI carbohydrate with greater portion sizes^(9)^. The present study has nearly 2·5 times higher sample size compared with a similar study that was done in 2020 in the country^(10)^. The aim of this study, therefore, was to investigate the association between dietary GI, GL and risk of breast cancer among Iranian women.

## Methods and materials

### Study population

This population-based case–control study was conducted on women aged > 30 years in Isfahan, Iran. A total of 350 women with incident breast cancer and 700 age- and sociodemographic-matched controls were recruited between July 2013 and July 2015. Convenience-sampling method was applied to select cases from those who were referred to hospitals or private clinics if disease status was diagnosed during the maximum of last 6 months by physical examination, mammography findings and pathological verification. We hypothesised that unhealthy dietary patterns would increase breast cancer risk by 1·5 times. Therefore, considering type I error of 5 %, the study power of 80 %, the common ratio of 0·25 and the ratio of controls to cases as 2, the required sample size was calculated to be 350 cases and 700 controls for this project. Among breast cancer patients (primary incident malignant breast tumours with invasive nature in medical records), those who had a prior history of surgical resection or chemotherapy or radiotherapy or all of them (this was for the current and their past conditions) were permitted to attend in our project. Patients with a history of any type of neoplastic lesion or cysts (except BC) and those with a prior history of any hormone replacement therapy were excluded. Cluster random sampling method was applied to select controls from apparently healthy women. Individuals who were not relatives of patients with BC that attended primary health care centres for their annual personal checkup or attended to receive required information about their children (i.e. growth monitoring, vaccination, …) were selected. From several healthcare centers in Isfahan, two centres were randomly chosen. First, considering the population under coverage, and then based on attendance of women to these centres, required sample was recruited. Controls were not included if they were of non-Iranian nationality, had a history of any cancer, cysts and pathological disease, followed special diets and had a history of hormone replacement therapy. Study participants who agreed to attend in our project, after informing about the study methodology, signed an informed consent form.

### Dietary intake assessment

Detailed dietary intake assessments have been described elsewhere^(11)^. In brief, dietary information of study participants was examined using a validated Willett-format semi-quantitative dish-based FFQ^(12)^ for Iranian population with 106 items about usual dietary intake over the past year. Trained nutritionists through face-to-face interviews with each participant, completed FFQ. Each participant was questioned about the usual consumption frequency of a specified portion of a given food in the preceding year (the last year before diagnosis of breast cancer for cases) on a daily, weekly or monthly basis. The interviewer was not blind to the health condition of study participants; however, the same FFQ was passed to both groups. The questionnaire comprised of five groups of foods and dishes as following: (1) mixed dishes (cooked or canned, twenty-nine items); (2) carbohydrate-based foods (different kinds of bread, cakes, biscuits and potato, ten items); (3) milk-derived products (dairies, butter and cream, nine items); (4) fruits and vegetables (twenty-two items) and (5) sundry food items and beverages (inclusive of sweets, fast foods, nuts, desserts and beverages, thirty-six items). For each food and mix dishes in the questionnaire, there was nine multiple-choice frequency response categories, which ranged from ‘never or less than once a month’ to ‘12 or more times per day’. The number of options in frequency response categories occasionally varied according to frequency consumption of food or dishes, for instance regarding seldom-used foods the number of multiple-choice options reduced, while for frequently used foods, more multiple-choice categories were considered. By considering the daily frequency and weight of the portion sizes of each food and mix dishes on the basis of household measures^(13)^, all the information was converted to food consumption in grams per day. Subsequently, the average daily intake of energy and nutrients was derived using US Department of Agriculture food composition database (Iranian modified version)^(14)^.

A classic validation study was not conducted for the current FFQ used in this study; however, given the significant expected associations between dietary data derived from this FFQ and several health-related outcomes in our previous publications^(15–18)^, it seems that the questionnaire works well in reflecting long-term dietary intakes.

### Calculation of dietary GI and GL

Total dietary GI values were computed by the following formula: ∑ (GI_a_ × available carbohydrate_a_)/total available carbohydrate, in which available carbohydrate was computed as total carbohydrate_a_ minus fibre_a_^(19)^. The US Department of Agriculture food composition table was the main source for extracting the total carbohydrate and fibre content of foods. Of the eighty-five carbohydrate-containing foods in our food list, the Iranian GI table^(20)^ provided GI values for only six foods. Thus, for the remaining food items, we used data from International glycaemic index tables^(21,22)^. Out of seventy-nine remaining food items, we found GI values for sixty-two foods in the International table. Glycaemic index values for seventeen food items were not available in that table; therefore, for these food items, we used the values for similar foods based on physical and chemical features^(23)^. All derived GI values from International glycaemic index tables were relative to glucose as the reference food however for values derived from Iranian GI table since the reference food was white bread we multiplied the amount of GI for each food by 0·7 to obtain the GI value based on glucose as the reference^(24)^. Regarding mixed meals and dishes, the GIs of individual food constituents were summed up^(19)^. The total dietary GL was calculated using the following formula: (total GI × total available carbohydrate)/100^(19)^.

### Assessment of breast cancer

Diagnosis of BC was performed on the basis of physical examination and mammography and complemented with pathological assessments. All patients with BC stages I–IV were qualified to take part in our project.

### Assessment of other variables

A pretested questionnaire was used to collect information about socio-demographic status (age, marital status, residence place and education), lifestyle information including alcohol consumption, smoking, medical information including disease history, menopause status, family history of BC, history of breastfeeding and supplement use in a face-to-face interview with each subject. A short form of the International Physical Activity Questionnaire^(25,26)^ was used to calculate the physical activity level of participants. Then, collected data were expressed as Metabolic Equivalent-hours per week. After measurement of anthropometric measures based on standard methods, each participant’s BMI was determined by dividing the weight (kg) by height squared (m^2^).

### Statistical analysis

Due to the weak correlation coefficients between dietary GI and GL and energy intake in the current dataset (*r* = 0·003 and 0·041, respectively), we did not use the residual method to adjust dietary GI/GL for energy intake. In other words, all analyses were done with non-adjusted values of dietary GI and GL for energy. To classify participants, tertile cut-points of dietary GI and GL were used. General characteristics of study participants across tertiles of dietary GI and GL were examined using one-way ANOVA for continues variables and chi-square for categorical variables. Comparison of dietary intakes across tertiles of dietary GI and GL was done using one-way ANOVA. The association of dietary GI and GL with breast cancer was assessed by using binary logistic regression in different models. Age (continuous) and energy intake (continuous) were considered in the first model. Then, we further controlled for educational status (educated/not educated), socio-economic status (poor/middle class/high class), place of residency (urban/rural), supplement use (yes/no), family history of breast cancer (yes/no), physical activity (continuous), marital status (married/not married/other), smoking status (smoker/non-smoker/ex-smoker), alcohol consumption (yes/no), history of breastfeeding (yes/no) and menopausal status (pre-menopause/post-menopause). BMI (continuous) was taken into account in the third model. In these analyses, the lowest intake of dietary GI and GL was considered as the reference category. The trend of odds ratios across increasing tertiles of dietary GI and GL was computed through considering the tertiles as an ordinal variable. In addition to the whole study population, the analyses were also done stratified by menopausal status. The statistical analyses were carried out by using SPSS (version 16). *P* values were considered significant at < 0·05.

## Results

General characteristics of study participants across tertiles of dietary GI and GL are presented in Table 1. Women in the highest tertile of dietary GI were less likely to be married compared with those in the lowest tertile. In addition, participants in the third tertile of dietary GL were more likely to live in urban areas than those in the first tertile. No other significant differences were identified in terms of other variables across tertiles of dietary GI and GL.

**Table 1:** General characteristics of study participants across tertiles of dietary GI and GL*

	Tertiles of dietary GI			*P*-value†	Tertiles of dietary GL			*P*-value†
	T_1_ (*n* 330)	T_2_ (*n* 338)	T_3_ (*n* 382)		T_1_ (*n* 338)	T_2_ (*n* 337)	T_3_ (*n* 375)	
	%	%	%		%	%	%	
Age (years)								
Mean	62·3	62·1	62·7	0·73	61·7	62·2	63·2	0·15
sd	11	10·7	10·7		11·2	10·8	10·4	
BMI (kg/m^2^)								
Mean	24·3	24·5	24·1	0·60	24·3	24·3	24·2	0·97
sd	5·2	5·3	5·2		5·4	5·2	5·1	
Physical activity (Met-h/week)								
Mean	34·8	35·3	34·9	0·54	35·5	34·5	35·1	0·19
sd	6·6	6·6	6·7		6·9	6·08	6·8	
Educated	28·5	22	25	0·15	28·1	25·8	21·6	0·12
Low SES	28·8	32·4	30	0·48	28·7	33·8	29·1	0·53
Urban-resided	38·2	36·3	33·9	0·49	60·9	60·2	69·9	0·01
Family history of breast cancer	4·8	6	5·5	0·81	3·8	6·2	6·1	0·29
Married	86·1	81·5	83·4	0·03	86·1	84·6	80·8	0·31
Current smoker	16·4	13·1	14·2	0·47	15·1	13·6	14·7	0·86
Alcohol consumption	6·1	6·8	6·6	0·91	5·9	7·4	6·1	0·69
Supplement use	9·1	8·6	11·8	0·29	9·2	8·9	11·5	0·44
History of breast feeding	31·8	36·3	33·4	0·46	34	35·6	32	0·59
Post-menopause	80	80·1	82·6	0·58	78·4	80·1	84·3	0·11

GI, glycaemic index; GL, glycaemic load; MET-h, Metabolic Equivalent-hours per week.

* All values are mean ± sd, unless indicated.

† ANOVA for continuous variables and *χ* ^2^ test for categorical variables.

Comparing dietary intakes across tertiles of dietary GI, we found that participants in the top tertile had higher intakes of cholesterol compared with those in the bottom tertile. No significant differences were observed among dietary intakes of study participants across tertiles of dietary GL (Table 2).

**Table 2:** Dietary intakes of study participants across tertiles of dietary GI and GL*

	Tertiles of dietary GI						*P*-value†	Tertiles of dietary GL						*P*-value†
	T_1_ (*n* 330)		T_2_ (*n* 338)		T_3_ (*n* 382)			T_1_ (*n* 338)		T_2_ (*n* 337)		T_3_ (*n* 375)		
	Mean	sd	Mean	sd	Mean	sd		Mean	sd	Mean	sd	Mean	sd	
Energy (kJ/d)	9660	2832	9330	3104	2669	2761	0·21	9388	2995	9614	2912	9660	2782	0·41
Nutrients														
Carbohydrate (g/d)	325	110	306	106	319	105	0·06	312	111	315	106	323	104	0·39
Protein (g/d	76·6	26·6	76	29·1	79·5	29·9	0·21	77	27·4	77·4	31·1	78·1	27·4	0·85
Fat (g/d)	84·1	32·1	83·8	39·9	85·6	32·7	0·77	82·1	32·7	87·18	39·2	84·47	32·6	0·17
Saturated fats (g/d)	32·2	24·7	32·4	29·3	31·7	25·6	0·93	30·5	22·7	33·9	31·6	31·7	24·6	0·24
MUFA (g/d)	20·4	7·8	19·8	9·01	20·5	8·1	0·47	19·89	7·9	20·81	8·8	20·22	8·1	0·34
PUFA (g/d)	9·81	5·9	9·78	8·9	10·4	6·1	0·34	9·56	5·2	10·3	9·3	10·2	6·1	0·30
Trans fats (g/d)	0·42	0·29	0·43	0·33	0·42	0·31	0·89	0·40	0·27	0·44	0·36	0·42	0·30	0·26
Cholesterol (mg/d)	184	105	181	100	201	125	0·03	190·39	101	187·1	115·2	190·8	116·7	0·89
Total fibre (g/d)	22·9	7·8	21·8	8·3	22·3	7·4	0·18	21·9	7·8	22·4	7·9	22·7	7·9	0·35
Vitamin A (mg/d)	3138	2404	3099	2855	3199	2760	0·88	3190	2542	3203	2927	3077	2599	0·78
Vitamin D (mg/d)	29	30·6	44·2	179	44	169	0·29	44·2	178·3	44·7	179	31·6	46·5	0·39
Vitamin E (mg/d)	6·44	3·44	6·22	3·38	6·73	3·43	0·14	6·16	2·98	6·63	3·78	6·62	3·45	0·12
Vitamin C (mg/d)	64·2	43·6	59·3	39·5	61·9	39·2	0·30	62·1	40	63·2	43·1	60·2	39·2	0·61
Vitamin B_6_ (mg/d)	1·62	0·50	1·58	0·56	1·62	0·52	0·54	1·59	0·50	1·61	0·56	1·62	0·52	0·70
Folate (mg/d)	287	97·1	270·3	102	282·3	103·8	0·08	279·9	108	274·9	96	284·2	89·9	0·47
Vitamin B_12_ (mg/d)	2·70	1·90	2·69	2·43	2·87	2·78	0·53	2·92	2·61	2·69	2·25	2·69	2·38	0·36
Potassium (mg/d)	2988·1	895	2834·7	967	2944·8	948	0·09	2916·5	968	2919·8	936	2932·8	913	0·97
Sodium (mg/d)	4836·8	1573	4796·5	1896	4934·8	1811	0·55	4739·1	1671	4883·6	1795	4937·9	1820	0·30
Zinc (mg/d)	10·26	3·17	9·95	3·30	10·32	3·23	0·27	10·08	3·09	10·14	3·37	10·31	3·24	0·61
Copper (mg/d)	1·72	0·54	1·65	0·61	1·70	0·53	0·22	1·68	0·58	1·67	0·52	1·71	0·58	0·69
Selenium (mg/d)	148·06	53·8	143·09	54·4	148·2	49·7	0·34	143·5	52·7	145·6	53·6	150	51·1	0·23
Iron (mg/d)	17·1	5·5	16·6	5·8	17·1	5·1	0·35	16·7	5·6	16·8	5·4	17·3	5·4	0·28
Magnesium (mg/d)	462·7	149·3	440·2	152·8	452·7	139·6	0·14	445·1	150·4	450·1	142·5	459·5	147·7	0·41
Food groups														
Fruits (g/d)	170·3	162·1	162·2	162·2	163	140·1	0·75	161·6	153·3	174·7	162·4	158·8	147·4	0·35
Vegetables (g/d)	82·8	63·9	76·5	69·9	82·8	82	0·42	83·3	77·2	82·7	76·7	64·5	3·3	0·44
Sea food (g/d)	5·38	8·75	8·74	35·6	9·85	38·3	0·14	8·21	34·9	9·46	38·03	6·99	18·06	0·57
Legumes (g/d)	14·25	11·45	15·64	20·38	14·42	11·82	0·42	14·73	13	15·02	19·55	14·60	11·65	0·93
Dairy (g/d)	235·1	153·7	219·5	136·5	238·3	171·5	0·23	238	150	235·7	166·5	221·9	149·4	0·31
Refined grains (g/d)	112·5	67·5	112·6	79	119	86·5	0·44	118·5	82·2	111·6	70	114·4	81·9	0·52
Whole grains (g/d)	329	161·6	308·5	154·9	313·6	148·3	0·20	305·4	158·4	317·1	150·8	326·9	154·4	0·18
Red and processed meats (g/d)	11·38	17·92	10·30	12·57	10·86	15·73	0·67	11·49	16·1	10·91	16·21	10·23	14·25	0·55
White meat (g/d)	65·43	61·89	74·07	77·49	78·31	4·31	0·07	72·27	4·15	74·06	84·74	72·60	66·22	0·94

GI, glycaemic index; GL, glycaemic load; MUFA, monounsaturated fatty acid; PUFA, polyunsaturated fatty acid.

* All values are mean ± SD.

† Obtained from ANOVA.

Crude and multivariable-adjusted OR and 95 % CI for breast cancer across tertiles of dietary GI and GL are indicated in Table 3. In the crude model, women in the top tertile of dietary GI had 40 % higher odds of breast cancer than those in the bottom tertile (OR: 1·40; (95 % CI 1·02, 1·91)). When we controlled for potential confounders including BMI, this association remained significant (OR: 1·47; (95 % CI 1·02, 2·12)). When we made stratified analysis by menopausal status, postmenopausal women in the highest tertile of dietary GI had 51 % greater odds for having breast cancer than those in the bottom tertile (OR: 1·51; (95 % CI 1·02, 2·23)). No significant association was found between dietary GI and odds of breast cancer among premenopausal women.

**Table 3:** Crude and multivariable-adjusted OR and 95 % CI for breast cancer across tertiles of dietary GI and GL*

	Tertiles of dietary GI					*P*-trend	Tertiles of dietary GL					*P*-trend
	T_1_	T_2_		T_3_			T_1_	T_2_		T_3_		
		OR	95 % CI	OR	95 % CI			OR	95 % CI	OR	95 % CI	
Breast cancer												
No. of cases	330	338		382			338	337		375		
Crude	1·00	1·03	0·74, 1·43	1·40	1·02, 1·91	0·02	1·00	0·97	0·70, 1·35	1·35	0·99, 1·84	0·05
Model I	1·00	1·11	0·78, 1·56	1·42	1·02, 1·97	0·03	1·00	0·90	0·64, 1·28	1·24	0·89, 1·72	0·17
Model II†	1·00	1·02	0·71, 1·46	1·39	0·99, 1·95	0·05	1·00	0·89	0·62, 1·27	1·17	0·83, 1·64	0·33
Model III	1·00	1·05	0·71, 1·53	1·47	1·02, 2·12	0·03	1·00	0·94	0·64, 1·38	1·24	0·86, 1·79	0·21
Premenopausal												
No. of cases	66	67		66			73	67		59		
Crude	1·00	1·07	0·47, 2·44	0·82	0·34, 1·95	0·66	1·00	0·80	0·36, 1·78	0·55	0·22, 1·33	0·74
Model I	1·00	1·12	0·47, 2·62	0·94	0·38, 2·30	0·90	1·00	0·84	0·37, 1·90	0·53	0·21, 1·33	0·74
Model II	1·00	0·91	0·36, 2·31	1·09	0·42, 2·81	0·86	1·00	0·67	0·27, 1·62	0·49	0·18, 1·34	0·70
Model III	1·00	1·05	0·34, 3·24	0·86	0·28, 2·66	0·92	1·00	0·92	0·32, 2·61	0·67	0·20, 2·24	0·54
Postmenopausal												
No. of cases	264	271		316			265	270		316		
Crude	1·00	1·02	0·71, 1·46	1·50	1·06, 2·11	0·01	1·00	1·00	0·70, 1·44	1·48	1·05, 2·08	0·01
Model I	1·00	1·10	0·75, 1·60	1·50	1·05, 2·14	0·02	1·00	0·93	0·64, 1·36	1·42	1·00, 2·03	0·03
Model II	1·00	1·01	0·68, 1·50	1·42	0·98, 2·06	0·05	1·00	0·91	0·61, 1·35	1·32	0·91, 1·91	0·12
Model III	1·00	1·03	0·68, 1·56	1·51	1·02, 2·23	0·03	1·00	0·94	0·62, 1·42	1·38	0·93, 2·05	0·08

GI, glycaemic index; GL, glycaemic load.

* All values are OR (95 % CI).

† Model II: additionally, adjusted for education, socio-economic status, urban-resided, supplement use, family history of breast cancer, physical activity, marital status, smoking status, alcohol consumption, breastfeeding and menopausal status.

Model I: adjusted for age and energy intake.

Model III: additionally, adjusted for BMI.

In terms of dietary GL, we found no significant association between dietary GL and odds of breast cancer either before (OR: 1·35; (95 % CI 0·99, 1·84)) or after adjustment for potential confounders (OR: 1·24; (95 % CI 0·86, 1·79)). Stratified analysis by menopausal status showed that postmenopausal women in the highest tertile of dietary GL had 48 % greater chance for having breast cancer compared with those in the lowest tertile (OR: 1·48; (95 % CI 1·05, 2·08)). This was the case when we controlled for age and energy intake (OR: 1·42; (95 % CI 1·00, 2·03)). However, the association became non-significant in the fully adjusted model (OR: 1·38; (95 % CI 0·93, 2·05)). No significant association was seen between dietary GL and odds of breast cancer among premenopausal women.

## Discussion

In this case–control study, we found a significant positive association between dietary GI and odds of breast cancer. Such association was also seen in postmenopausal women. However, we failed to find any significant association between dietary GL and odds of breast cancer.

Breast cancer, as one of the most important health issues, globally consumes substantial health resources, which in turn would inflict an enormous health burden in future^(27)^. Decreased age at the onset of this malignancy highlights the need for more attention to deal with this problem^(28,29)^. Among modifiable lifestyle factors, diet plays an important role in the pathogenesis of breast cancer^(30)^. We observed a positive association between dietary GI, but not dietary GL, and odds of breast cancer. In line with our findings, a recent case–control study on 136 Iranian breast cancer survivors showed a significant association between dietary GI and odds of breast cancer; however, no significant association was found for dietary GL^(10)^. Similarly, a recent meta-analysis on global data from 185 prospective studies (published to April 30, 2017) and 58 clinical trials (published to Feb 28, 2018) with 4635 adult participants showed a significant association between dietary GI, but not dietary GL, and breast cancer among postmenopausal women^(31)^. However, several studies that examined dietary GI/GL in relation to the risk of breast cancer showed no significant association^(32–34)^. A modest non-significant association of GI/GL and breast cancer have also been suggested by Turati *et al*. in a systematic review and a series of meta-analyses which included original cohort or case–control studies up to May 2019^(35)^. Numerous studies have provided evidence on the increased risk of breast cancer with increased consumption of foods with high GI and GL, or GL alone^(6,36,37)^. The inconsistencies between our findings and those of earlier studies might be explained by several reasons: (1) different study designs: some studies^(6,33,34)^ with prospective design were undertaken to obtain required insight in this regard; whereas our work was a case–control study; (2) different sample size: Amadou *et al*.^(32)^ in a similar study design has assessed this association on 1000 cases and 1074 matched control women; while our study was done on 350 cases and 700 control subjects; (3) different methodology: considering confounding effects of the overall diet on GI and GL, Woo *et al*.^(36)^ in their study applied more holistic approach to assess GI/GL in relation to breast cancer risk. In other words, they applied the reduced rank regression method to identify high GI and GL dietary pattern, rather than considering dietary GI and GL alone as an independent variable and (4) different dietary patterns: the dietary GI/GL differs between different societies.

In our study, a significant positive association was observed between dietary GI and odds of breast cancer among postmenopausal, but not premenopausal women. With regard to dietary GL, we found no significant association between dietary GL and odds of breast cancer among either pre- or postmenopausal women. Our findings were in agreement with a prospective Canadian National Breast Screening Study, conducted by Navarro Silvera and colleagues on 1461 women diagnosed with breast cancer. In that study, the RR for breast cancer in postmenopausal women comparing extreme tertiles of GI was 1·87 (95 % CI 1·18, 2·97)^(7)^. A positive association between dietary GI and GL and breast cancer in postmenopausal women was also documented by two case–control studies^(10,36)^. In contrast to our findings, in a recent meta-analysis, postmenopausal women with higher dietary GL had higher risk of breast cancer^(38)^. Two prospective cohort studies have failed to find any significant association between dietary GI and GL and risk of breast cancer among postmenopausal women^(6,8)^. Consistent with ours, previous published data showed no association between dietary GI/GL with breast cancer risk among premenopausal women^(6,32,38)^. However, some studies reported a positive association between dietary GI or GL and risk of breast cancer among premenopausal women^(36)^. The possible reasons for the different associations in pre- and postmenopausal women have been provided below.

Some hypotheses have been proposed on how high-GI/GL diets might elevate the risk of breast cancer. First, elevated levels of insulin after high glycaemic meals might provide a reason. Given the biological function of insulin in the liver as down-regulating the synthesis of sex hormone-binding globulin and insulin-like growth factor (IGF-1) – binding proteins 1, increased bioavailability of both sex hormones and IGF-1 is evident^(39–41)^. Proliferative property of these factors in conjunction with cell differentiative and antiapoptotic actions of IGF-1^(41,42)^ create metabolic environment which is susceptible for breast tumour growth. In addition, dietary GI and GL may be involved in pathways that affect concentrations and composition of serum lipids, C-reactive protein and other markers of inflammation which has long been proposed in the pathology of breast cancer^(43)^. Finally, to justify potential interaction of menopause status with breast cancer risk, oestrogen depletion is a trigger for increased insulin resistance in postmenopausal women and as a result impairment of glucose transport into the cells is expected^(44)^. However, in our study, the increased risk among postmenopausal women was limited to dietary GI, not GL. In trying to shed light on this difference, it is worth mentioning that overall dietary GI is indicative of overall carbohydrate quality in the diet, whereas total dietary GL that was structured based on the amount of carbohydrate consumed, captures both the quality and quantity of total dietary carbohydrate intake^(45)^.

In our study, median values of dietary GI were 62·9 ± 2·4 for control subjects and 63·2 ± 2·4 for cases. In terms of dietary GL, these values were equal to 179·3 ± 63·9 for controls and 186·9 ± 66·6 for cases. In a Mexican population-based case–control study, European Prospective Investigation into Cancer and Nutrition and Melbourne Collaborative Cohort Study median values of dietary GI/GL were lower than those in our study. In a hospital-based case–control study in Italy, median values of dietary GI were higher, and those of dietary GL was lower than those of our study. The difference between values of dietary GI/GL in different studies originates from the different food patterns across different communities which may justify inconsistent findings across studies.

Several strengths of this study are noteworthy. We had the chance of performing a large population-based study. Another strength was given by controlling for a wide range of confounders, therefore, an independent association between dietary GI/GL and odds of breast cancer was expected. Finally, the possibility of changing usual dietary intakes of participants has been reduced by enrollment of new cases of breast cancer. However, interpretation of our findings should be done in the context of potential limitations. The main limitation is given by the nature of case–control study such that we cannot confer causality. In addition, as with all case-control studies, we cannot rule out the possibility of selection and recall bias. Due to the nature of FFQ, it is difficult to exclude the impacts of subject’s misclassification on the final results. Despite controlling for several confounders, the possibility of residual confounding still remains. One might question the lack of controlling for dietary fibre intake in our study. It must be kept in mind that dietary GI, GL and dietary fibre are all concepts related to carbohydrates. We did not control the GL models for dietary fibre because the fibre content of foods can affect its GI and therefore GL values. Therefore, adjustment for dietary fibre intake would somehow be an overadjustment.

In this case–control study, the interviewer was not blind to the health condition of individuals because cases were interviewed in the clinical setting of healthcare office or private clinics while controls were interviewed in other places; however, the interviewer was trained to use similar questions for both groups. The included cases were not representative of breast cancer cases in general since we recruited patients from hospitals or private clinics through convenience sampling method (each patient in the hospital or private clinic that met the inclusion criteria was included in the study) and no random sampling was done in this regard.

In conclusion, the findings of this study revealed a significant positive association between dietary GI and the odds of breast cancer. However, we observed no significant association between dietary GL and odds of breast cancer.

## Supplementary material

For supplementary material accompanying this paper visit https://doi.org/10.1017/S1368980021004018. click here to view supplementary material
